# Contained Jejunal Perforation Secondary to Ingestion of Magnet Balls in a Nonfatal Drowning Child

**DOI:** 10.7759/cureus.29741

**Published:** 2022-09-29

**Authors:** Osama Kattih, Zainab A Almoosa, Abdulazeem Alibraheem

**Affiliations:** 1 Pediatric Intensive Care Unit, Almoosa Specialist Hospital, Alahsa, SAU; 2 Pediatric Infectious Diseases, Almoosa Specialist Hospital, Hofuf, SAU; 3 Pediatric Gastroenterology, Maternity and Children Hospital in Alhasa, Hofuf, SAU

**Keywords:** peritonitis, pediatric emergencies and critical care, jejunal perforation, magnets ingestion, pediatric gastroenterology

## Abstract

Most ingested foreign bodies often pass through the gastrointestinal tract uneventfully; however, complications such as perforation do occur. Jejunal perforation is a known complication of abdominal trauma. While gastrointestinal tract injury due to the ingested foreign body such as a toothpick, fishbone, and battery among others are common, jejunal perforation is not very common in pediatrics.

We report an unusual case of jejunal perforation that was diagnosed after a child was admitted to the pediatric intensive care unit for nonfatal drowning. A 15-month-old girl presented to our emergency room after she was found submerged in a swimming pool. She was unwitnessed for about ten minutes. At the scene, she was apneic, and cyanotic but had a pulse. Cardiopulmonary resuscitation was started and she was brought to our emergency room. She was managed for her drowning injuries and was accidentally found to have a foreign body on her abdomen by x-ray. She had no signs or symptoms of perforation, however, after questioning the parents they told us that they brought her a toy containing magnet balls about one month ago. After stabilizing her respiratory status and correcting her acidosis, an upper gastrointestinal (GI) endoscopy was done that showed jejunal perforation and multiple magnets. A consultation was done immediately for the pediatric surgery team then the perforation was repaired. High-powered magnets represent a serious health hazard if ingested due to risks of gastrointestinal perforation. It is important to have a high index of suspicion for potential injuries, especially silent ones. One of the rare complications is contained jejunal perforation.

## Introduction

Since 2002, there has been a significant increase in the incidence of magnetic foreign body injuries [1]. The incidence of pediatric magnet ingestions and subsequent interventions has increased over time [2]. Data collected from the National Electronic Injury Surveillance System database showed an alarming 8.5-fold increase in the incidence of magnetic ingestions in children between 2002 and 2011 [3].

Magnets have a high risk of requiring surgical intervention for removal. About 1% of cases require surgical treatment for foreign body retention or associated complications [4]. One retrospective analysis from a single-center experience from China published in 2021 showed the majority of magnet ingestion cases were asymptomatic [4]. In another study published by The American Academy of Pediatrics in March 2022 about 46% of children’s hospital patients who ingested magnets needed endoscopy and/or surgery [5]. High-powered magnets composed of neodymium are now common components of household appliances and some toys. They represent a serious health hazard if ingested due to risks of gastrointestinal perforation [6]. Two or more high-powered magnets, especially if ingested at different times, may attract across layers of the bowel leading to pressure necrosis, fistula, volvulus, perforation, infection, or obstruction; this may result in serious consequences including intestinal resection. It is postulated that intraperitoneal hemorrhage could occur if mesenteric vessels are trapped between attracted bowel loops [4]. Although ingestion of a single magnetic foreign body (FB) may, in most cases, be managed as simple foreign body ingestion, the ingestion of multiple magnetic FB is associated with a high risk of complications and requires aggressive management [7]. Jejunal perforation can be either free or contained. Free perforation arises when bowel contents leak freely into the abdominal cavity and cause diffuse peritonitis. Contained perforation occurs when the ulcer creates a full-thickness hole, but free leakage is prevented by contiguous organs such as the pancreas or omentum that wall off the area. Because of the wrapping of the omentum after perforation or the formation of an internal fistula directly, the clinical symptoms are mild and the imaging findings are not typical [4]. There are no reported cases of jejunal perforation after nonfatal drowning in the literature, but there is one reported case of unwitnessed magnet ingestion in a five-year-old boy leading to bowel perforation after magnetic resonance imaging [8].

## Case presentation

**Figure 1:**
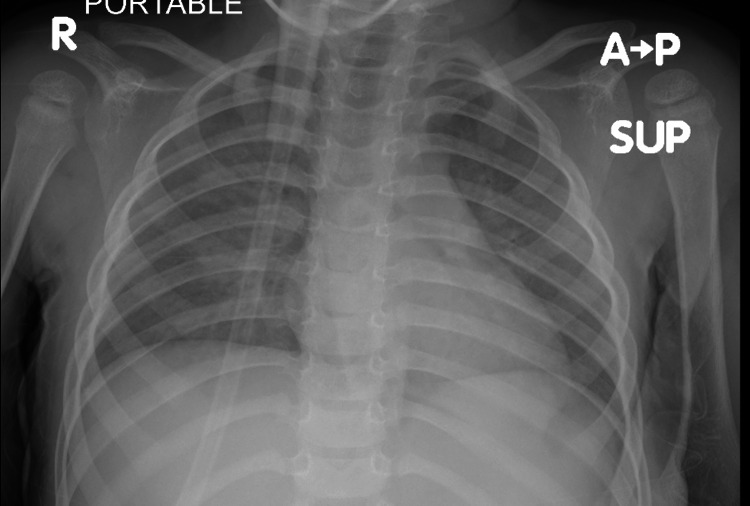
Chest x-ray

A 15-month-old girl previously healthy was with her parents at a farm with a swimming pool, she was unwitnessed for about ten minutes, then she was found in the swimming pool face down in the pool unresponsive, she was pulled out of the water and cardiopulmonary resuscitation was started. She had a pulse of 80/min but she was not breathing and cyanotic with water coming out of her mouth and nose. Mouth-to-mouth resuscitation was done and she was brought to our emergency room. She arrived at the emergency room 15 min after she was resuscitated at the scene, she was connected to a cardiopulmonary monitor; her temperature was 36°C, cyanotic with an O2 saturation of 80% on room air, tachypneic with a respiratory rate of 36/min, tachycardic with heart rate (HR) of 142/min and hypertensive with blood pressure (BP) of 129/77. First arterial blood gas showed: potential hydrogen (PH) 7.12, partial pressure of carbon dioxide (PCO2) 42, partial pressure of oxygen (PO2): 52, Bicarb: 13.3, base excess (BE) -16, Lactate: 6.7, O2 Sat 75%. Immediately oxygen was started by a non-rebreather mask, and oral and nasal suction was done. A moderate amount of fluid was suctioned and her O2 saturation went up to 95%. She was given worm normal saline bolus of 20 ml/kg. A chest x-ray was done it showed bilateral infiltration (Figure 1).

**Figure 2:**
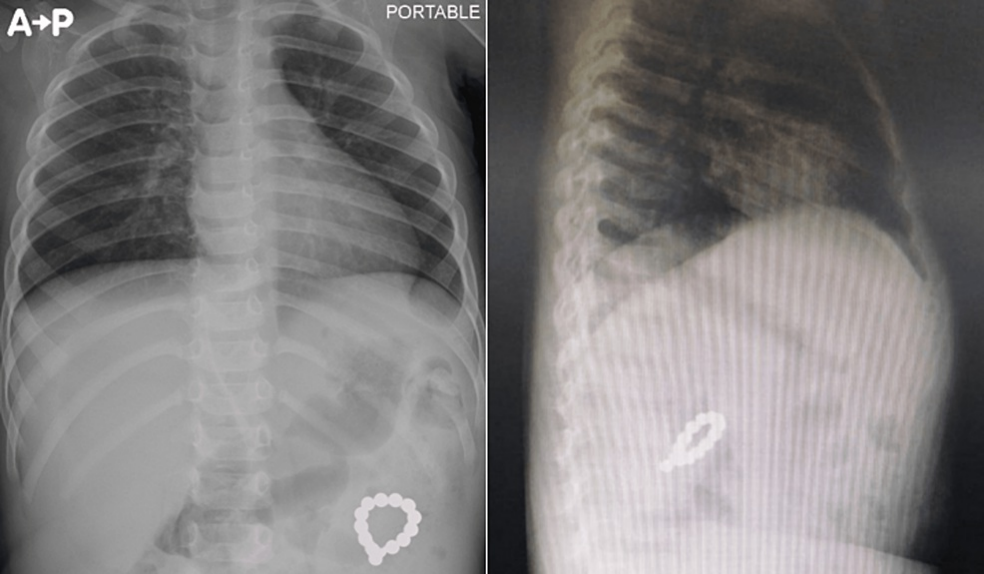
Repeat Chest x-ray

After stabilization, she was transferred to the pediatric intensive care unit (PICU) for further management. In the PICU supportive care was continued with oxygen and intravenous (IV) fluid, repeat arterial blood gas was done and it showed improvement with PH 7.32, PCO2 34, PO2: 93, Bicarb: 17.5, BE -8.6, Lactate: 1.1, O2 Sat 96.1%. A repeat chest x-ray was done and showed improvement in her lungs however there was a ring-like structure of a radiopaque foreign body in the left upper quadrant of the abdomen. Two views of the abdominal x-ray were done (Figure 2) demonstrating the same findings with no other foreign bodies.

**Figure 3:**
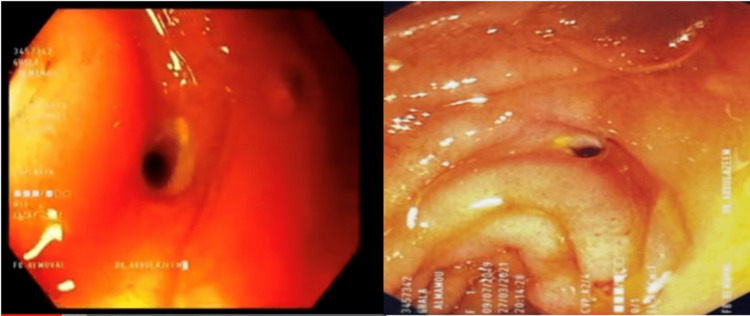
Endoscopy Photos

She was previously healthy with no signs or symptoms of gastrointestinal problems, however on reviewing with the parents, they recalled the recent purchase of a magnetic toy for the child with similar-looking metallic beads about one month ago. The gastroenterology team was called for evaluation and decided to do upper endoscopy as soon as she is stable. Her acidosis was corrected and her lactate went down from 6.7 to 1.1 and her oxygen was weaned off gradually to room air and she was stabilized after 10 hours of the submersion. She was taken to the operation room (OR) for an upper gastrointestinal (GI) endoscopy. This revealed a ring of magnetic balls stuck together with one of them attached to the intestinal mucosa but came off easily with a clear perforation (Figure 3) just after passing the duodenojejunal flex.

**Figure 4:**
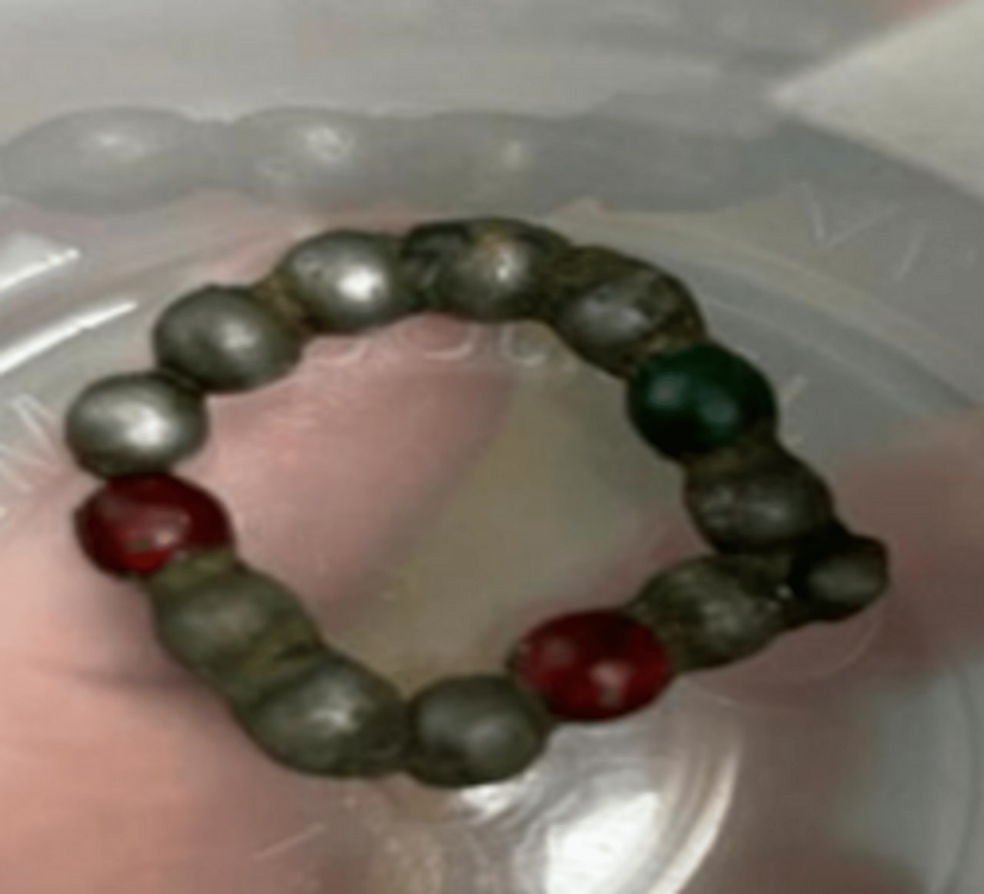
Actual magnet balls removed from the patient

**Figure 5:**
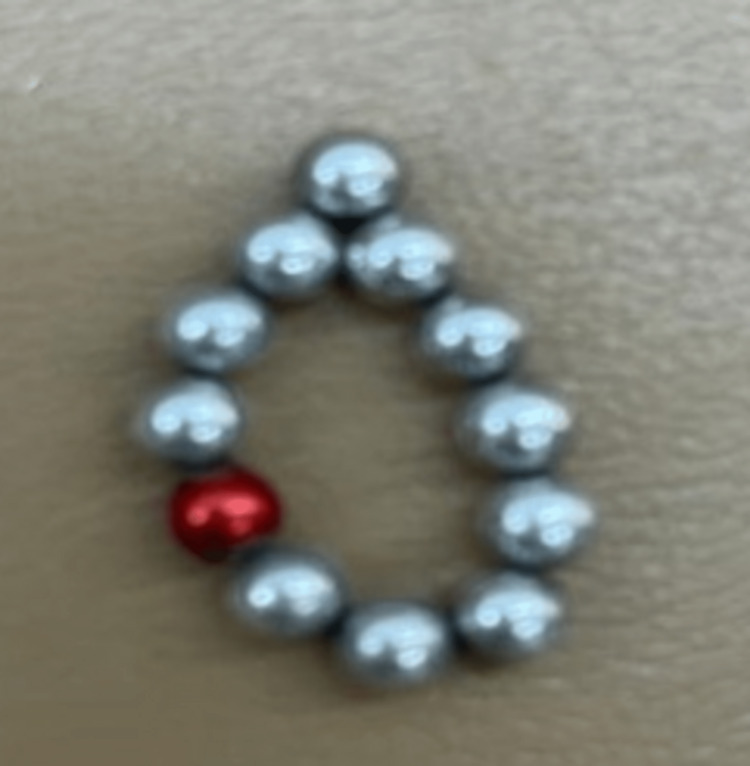
Magnet balls toy from home

The pediatric surgery team was called and an exploratory laparotomy was done. The small intestine was explored where they found perforation of the proximal loop of the jejunum, 12 cm from treitz angle, and through this perforation, they removed 14 magnets (Figure 4). Similar magnet balls were brought from home by parents similar to the ones removed (Figure 5). The perforation was sutured in a double layer. The exploration of the anterolateral aspect of the duodenum did not show any abnormality or perforation.

A moderate amount of fluid was suctioned from the peritoneal cavity and cultures were sent before the abdomen was closed. She was brought back to the PICU, she was extubated to oxygen per nasal cannula, and gradually weaned off. Her peritoneal fluid cultures grew Enterobacter cloacae and Klebsiella pneumonia, she was treated for peritonitis with ten days of metronidazole and piperacillin/tazobactam and was discharged home in stable condition. Multiple follow-up visits demonstrated a normal healthy child with no sequelae or complications.

## Discussion

Most foreign bodies, if small enough, are managed conservatively with watchful waiting and serial x-ray images. Fortunately, 80% of cases will usually have a spontaneous passage of the foreign body, with 10% to 20% requiring endoscopic removal and 1% requiring surgical intervention. Our case is a very unusual case of contained jejunal perforation secondary to the ingestion of magnet balls that was accidentally discovered in a nonfatal drowning child. In our case, the ingestion was unwitnessed by parents, leading to delayed diagnosis and complications of jejunal perforation.

Delayed diagnosis is a common concern. Another hindrance to effective management is that the initial diagnostic modality of x-ray imaging may mislead in exactly locating the magnetic foreign bodies, especially in asymptomatic patients. After resuscitation and correction of her hypoxia and acidosis, she was found to have contained jejunal perforation secondary to magnet balls. Even though she was asymptomatic maybe she started to have pain secondary to a small amount of leaking or peritonitis that lead to drowning. In addition hypoxia and acidosis secondary to drowning can contribute to her jejunal perforation. The main reason for her morbidity is for sure the ingestion of magnet balls that either led to drowning or perforation. Magnetic forces of attraction inside visceral organs can be devastating. They allow the magnetic foreign bodies to attract one another, in between bowel loops. Once the two loops are in contact, pressure necrosis ensues, resulting in ischemic injury and subsequent perforation.

Our case highlights the importance of prompt diagnosis and intervention to prevent complications. Greater physician awareness is also essential to suspect ingestion even in the presence of relatively non-specific symptoms or even completely asymptomatic.

## Conclusions

Foreign body ingestion is common in pediatrics, but high-powered magnets represent a serious health hazard if ingested due to risks of gastrointestinal perforation. Even asymptomatic children can have magnets ingested that could cause serious problems later, and if left untreated could cause either free or contained perforation.

It is important to have a high index of suspicion for potential injuries, especially silent ones, and to ask parents for risk factors of foreign body ingestion. For years, The American Academy of Pediatrics has been warning families about the dangers of high-powered magnets and urging lawmakers to take action. The American Academy of Pediatrics recommends families not keep high-powered magnet sets in their homes. Those who do have them should keep them in a locked container where children can’t get to them. Parents who suspect their child swallowed a magnet should seek immediate medical attention.
